# Molecular Mechanisms Mediating the Adaptive Regulation of Intestinal Riboflavin Uptake Process

**DOI:** 10.1371/journal.pone.0131698

**Published:** 2015-06-29

**Authors:** Veedamali S. Subramanian, Abhisek Ghosal, Rubina Kapadia, Svetlana M. Nabokina, Hamid M. Said

**Affiliations:** 1 Department of Medicine, University of California, Irvine, California, United States of America; 2 Department of Physiology/Biophysics, University of California, Irvine, California, United States of America; 3 VAMC, Long Beach, California, United States of America; University of Missouri-Kansas City, UNITED STATES

## Abstract

The intestinal absorption process of vitamin B2 (riboflavin, RF) is carrier-mediated, and all three known human RF transporters, i.e., hRFVT-1, -2, and -3 (products of the *SLC52A1*, *2 & 3* genes, respectively) are expressed in the gut. We have previously shown that the intestinal RF uptake process is adaptively regulated by substrate level, but little is known about the molecular mechanism(s) involved. Using human intestinal epithelial NCM460 cells maintained under RF deficient and over-supplemented (OS) conditions, we now show that the induction in RF uptake in RF deficiency is associated with an increase in expression of the hRFVT-2 & -3 (but not hRFVT-1) at the protein and mRNA levels. Focusing on hRFVT-3, the predominant transporter in the intestine, we also observed an increase in the level of expression of its hnRNA and activity of its promoter in the RF deficiency state. An increase in the level of expression of the nuclear factor Sp1 (which is important for activity of the *SLC52A3* promoter) was observed in RF deficiency, while mutating the Sp1/GC site in the *SLC52A3* promoter drastically decreased the level of induction in *SLC52A3* promoter activity in RF deficiency. We also observed specific epigenetic changes in the *SLC52A3* promoter in RF deficiency. Finally, an increase in hRFVT-3 protein expression at the cell surface was observed in RF deficiency. Results of these investigations show, for the first time, that transcriptional and post-transcriptional mechanisms are involved in the adaptive regulation of intestinal RF uptake by the prevailing substrate level.

## Introduction

Riboflavin (RF) a member of the family of water-soluble vitamins, is indispensable for normal human health due to its essentiality for cellular metabolism, proliferation, growth and survival. In its coenzyme forms, i. e., flavin mononucleotide (FMN) and flavin adenine dinucleotide (FAD), the vitamin plays key metabolic roles in biological oxidation-reduction reactions involving carbohydrate, lipid, amino acid, and certain vitamins (vitamin B_6_ and folate) [1]. A role for RF in protein folding in the endoplasmic reticulum has also been identified [2]. More recent studies have identified anti-oxidant [3–5] and anti-inflammatory [6] properties for RF as well as a role in normal immune function [7, 8], and in the maintenance of normal intestinal homeostasis [9]. Thus, disturbance in normal RF homeostasis is expected to lead to negative health consequences, which include degenerative changes in the nervous system, anemia, cataract and growth retardation [10, 11]. RF deficiency/suboptimal levels occur in a variety of conditions that include chronic alcoholism [12, 13], inflammatory bowel disease [14, 15], inborn errors of RF metabolism [infantile Brown-Vialetto Van Laere (BVVL) and Fazio Londe syndromes; autosomal recessive disorders caused by mutations in hRFVT-2 & -3] [16–19], and diabetes mellitus [20]. In contrast to the negative effects of RF deficiency, optimizing RF body homeostasis is effective in the treatment of patients with BVVL [16–19], and those with RF-responsive multiple acyl-CoA dehydrogenase deficiency [21, 22]; it also appears to have the potential to protect vital tissues from ischemia-induced oxidative injury [23].

Humans and other mammals (which lack the ability to synthesize RF endogenously) obtain the vitamin from exogenous sources via intestinal absorption. Previous studies have shown that the small intestinal and colonic RF uptake process is specific and carrier-mediated, and that all the three recently cloned RF transporters (RFVT-1, -2 & -3; products of the *SLC52A1*, *SLC52A2* & *SLC52A3* genes, respectively) are expressed in the human gut, with expression of RFVT-3 being the predominant [11, 24–28]. Other studies [26] have utilized live cell confocal imaging to show that the expression of RFVT-3 is restricted to the apical membrane domain of polarized intestinal epithelial cells, while that of RFVT-1 is mainly at the basolateral membrane domain (BLM); expression of hRFVT-2 was mostly in intracellular vesicular structures with some at the BLM [26]. An important role for RFVT-3 in intestinal RF absorption process has also been shown in studies utilizing gene-specific silencing approach (siRNA) [26].

Previous studies from our laboratory have shown that the intestinal RF uptake process is adaptively-regulated by the prevailing vitamin level [29–31]. Utilizing rats fed RF deficient and over-supplemented (OS) diets as well as human intestinal epithelial (Caco-2 and NCM460) cells maintained under RF deficient and OS conditions, a significant induction in intestinal RF uptake was observed in deficiency while suppression was observed upon OS [29–31]. Similar adaptive regulation in RF uptake by human renal and retinal pigment epithelial cells in RF deficiency was seen [32, 33], suggesting a global nature of this adaptive response. Little is known about the molecular mechanism(s) involved in mediating this adaptive response in intestinal RF uptake process. Taking advantage of the recent availability of appropriate molecular tools, we investigated this issue in this study. Our results show, for the first time, that the adaptive regulation in intestinal RF uptake process by substrate level is mediated at both the transcriptional and post-transcriptional levels.

## Materials and Methods

### Materials

Human-derived intestinal epithelial NCM460 cells were obtained from INCELL (San Antonio, TX). [^3^H]-RF (specific activity: 21.2 Ci/mmol, radiochemical purity: > 98%) was purchased from Moravek Biochemicals (Brea, CA). Anti-hRFVT-1 polyclonal antibodies obtained from Abnova (Walnut, CA). Anti-Sp1, anti-hRFVT-2, anti-hRFVT-3 and anti-β-actin monoclonal antibodies were obtained from Santa Cruz Biotechnology (Santa Cruz, CA). Anti-rabbit IRDye-800 and anti-mouse IRDye-680 antibodies were purchased from LI-COR Bioscience (Lincoln, NE). All cell culture supplies, transfection and molecular biology reagents were obtained from commercial vendors.

### Cell culture and RF uptake assay

For regular maintenance, the NCM460 cells were grown in F-12/Ham medium (Life Technologies Inc., MD) supplemented with 20% FBS, glutamine (0.29 g/l), penicillin (100,000 U/l), and streptomycin (10 mg/l). To examine the effect of extracellular RF level on [^3^H]-RF uptake, protein and RNA expressions, Firefly Luciferase mRNA expression, and histone modification, the cells were grown for 14 days in custom-made RF deficient (~0μM RF) and RF over-supplemented (OS; 100μM RF) DMEM (Life Technologies Inc.,) with 2.5% dialysed FBS (Hyclone) growth media were changed every 3 days. For [^3^H]-RF uptake, NCM460 cells were grown in regular 12 well plates and cells were incubated in Krebs-Ringer (K-R) buffer as described before [26, 34]. Protein content in the samples was determined in parallel wells using a Bio-Rad D_C_ protein assay kit (Bio-Rad, CA).

### Western blot analysis

Cell lysates were isolated from RF deficient and OS NCM460 cells and separated in NuPAGE 4–12% Bis-Tris gradient minigels (Invitrogen), transferred onto immobilon polyvinylidene difluoride membrane (PVDF) (Fisher Scientific). The anti-hRFVT-1, -2, -3, Sp1 and anti-β-actin antibodies were used as primary antibodies. The secondary antibodies used were anti-rabbit IRDye-800 and anti-mouse IRDye-680 (both at 1:30,000 dilutions). The specific immunoreactive bands were detected using the Odyssey infrared imaging system (LI-COR Bioscience, Lincoln, NE) and their densities were quantified using the LI-COR software.

### Semi-quantitative RT-PCR analysis

One microgram of total RNA isolated from NCM460 cells was treated with DNase I and reverse transcription was performed using iScript cDNA synthesis kit (Bio-Rad, CA). The hRFVT-1, -2, -3 and β -actin mRNA expression levels were determined by PCR amplification using Taq DNA polymerase enzyme (Clontech, CA), and gene specific primers (Table 1). The PCR conditions were used as previously described [34, 35], and data was normalized relative to β-actin expression in the same sample.

**Table 1:** Combination of primers used to amplify coding of the respective genes by real-time and semi-quantitative PCR.

Gene Name	Forward and Reverse Primers (5’-3’)
Semi-quantitative RT-qPCR primers	
hRFVT-1	AAAAGACCTTCCAGAGGGTTG;AGCACCTGTACCACCTGGAT
hRFVT-2	CCCTGGTCCAGACCCTA;ACACCCATGGCCAGGA
hRFVT-3	CCTTTCCGAAGTGCCCATC;AGAAGGTGGTGAGGTAGTAGG
β-actin	AGCCAGACCGTCTCCTTGTA;TAGAGAGGGCCCACCACAC
Real-time PCR primers	
Human Sp1	CCATACCCCTTAACCCCG;GAATTTTCACTAATGTTTCCCACC
β-actin	AGCCAGACCGTCTCCTTGTA; TAGAGAGGGCCCACCACAC
Firefly luciferase	CTCACTGAGACTACATCAGC; TCCAGATCCACAACCTTCGC
Renilla luciferase	GGAATTATAATGCTTATCTACGTGC; CTTGCGAAAAATGAAGACCTTTTAC
hnRNA primers	
*SLC52A3*	TCTCAGCACTTGGCTTTATC; CTCCCATGCGTATGTATGTA
β-actin	TTCCTGGGTGAGTGGAG; GGACTCCATGCCTGAGAG
ChIP assay and RT-qPCR	
*SLC52A3*	GGGTTCGCTCAGTGAAGGTA;CACAACGGACACACTCCTGCT

### Heterogeneous nuclear RNA (hnRNA), SLC52A3 promoter construct transfection and quantitative Real-time PCR (RT-qPCR) analysis

Total RNA isolated from NCM460 cells maintained under RF deficient and OS conditions for 14 days were used to perform RT-qPCR with *SLC52A3* gene specific hnRNA primers that anneals to sequences in the intron [36, 37]. RNA samples were treated with DNAse I and reverse transcribed (Bio-Rad, CA). RT-qPCR was performed using the *SLC52A3* gene specific hnRNA primers (Table 1). In parallel, a negative control without cDNA template was run with every PCR reaction to establish specificity. The human Sp1 and β-actin mRNA expression levels were determined by RT-qPCR amplification using gene specific primers for human Sp1 (Table 1). The RT-qPCR was performed as described previously [26, 35] and data normalized to β-actin were calculated using a relative relationship method [38].

The wild-type and mutated *SLC52A3* minimal (-199/+8) promoter constructs were generated and characterized previously [39]. NCM460 cells maintained in RF deficient and OS media for 7 days and then seeded into 12 well plates and cells were co-transfected with 3 μg/well of *SLC52A3*–199/+8 or mutated promoter constructs along with 100 ng of the *Renilla* transfection control plasmid *Renilla* luciferase-thymidine kinase (pRL-TK) (Promega) using Lipofectamine 2000 reagent (Life Technologies). After transfection, cells were maintained in respective growth medium for additional 7 days before RNA was isolated. The qRT-PCR was performed using respective cDNAs, Firefly Luciferase and Renilla Luciferase gene specific primers (Table 1). We examined promoter activity by determining the Firefly Luciferase mRNA expression levels which were normalized relative to the Renilla Luciferase mRNA expression in NCM460 cells maintained in RF deficiency and OS conditions. This was due to interference of the RF color (yellow) with the Luminometer reading.

### Chromatin immunoprecipitation (ChIP) assay and RT-qPCR

ChIP analysis was performed using the simple ChIP enzymatic chromatin IP kit (Cell Signaling, Inc.,) as per the manufacturer's instructions [40]. Briefly, NCM460 cells (4 × 10^6^ cells) grown for 14 days in RF deficient and OS media were washed with PBS. Proteins and DNA were then cross-linked with a final concentration of 1% formaldehyde at room temperature (RT) for 10 min. The reaction was stopped by the addition of glycine stop solution (10x) and incubated for 5 min at RT. After washing cells with ice-cold PBS, 2 ml of ice-cold PBS containing protease inhibitor cocktail was added, cells were scraped and centrifuged at 1500 rpm for 5 min at 4°C. The cells were then resuspended in 1 ml ice-cold buffer A containing DTT and PIC and incubated on ice for 10 min, and the nuclei were prepared as per the manufacturer instructions. Following that, the chromatin was digested with micrococcal nuclease, sonicated to shear DNA into fragments, and the samples were clarified by centrifugation at 10,000 rpm for 10 min at 4°C. The supernatant containing the cross-linked chromatin preparation was incubated for overnight with 1–2 μg of the specific antibody [H3, H3K4me3, H3K27me3 and IgG (Millipore)]. After incubation, samples were subjected to DNA purification following manufacturer’s protocol. Finally, the purified DNA was analyzed by RT-qPCR using *SLC52A3* minimal promoter (-199/+8) specific primers (Table 1). The PCR conditions used were denaturation at 95°C for 5 min, followed by 30 cycles of denaturation at 95°C for 30 s, annealing at 58°C for 15 s, and extension at 72°C for 15 s. The qPCR data was normalized to percent input and represented as percent enrichment relative to H3 and compared to control as described before [41]. No amplified product was detected in IgG negative control.

### Cell surface biotinylation

NCM460 cells maintained for 14 days in RF deficient and OS media were biotinylated using an EZ-Link Sulfo-NHS-Biotinylation Kit (Thermo Fisher Scientific Inc., Rockford, IL) and then lysed. In order to isolate biotinylated proteins, an equal amount of total soluble protein from NCM460 cells maintained in RF deficient or OS media was then incubated with avidin-conjugated agarose. The agarose-avidin beads were washed three times following manufacturer’s protocol and the bound biotinylated protein eluted by heating with DTT at 95°C. The eluted protein samples were subjected to western blot analysis in a NuPAGE 4–12% Bis-Tris gradient minigel (Invitrogen). For normalization, an equal protein concentration of total NCM460 cell lysate was loaded simultaneously and the level of surface expression of hRFVT-3 was determined using hRFVT-3 specific primary antibodies. After washing with PBST the blots were probed with IRDye 800 labeled goat-anti rabbit (1:30,000) secondary antibody. Specific bands for hRFVT-3 were detected and quantified as described previously.

### Data presentation and statistical analysis

RF uptake data represented in this paper are the result of at least three separate experiments and are expressed as mean ± SE in fmol/mg protein/5 min. Western blot, PCR, promoter, histone modifications, and biotinylation assays all were performed on at least three or more separate occasions. Data were analyzed by the Student's *t*-test with statistical significance set at P < 0.05.

## Results

### Effect of RF deficiency and OS on molecular parameters of RF uptake by human intestinal epithelial NCM460 cells

**Fig 1:**
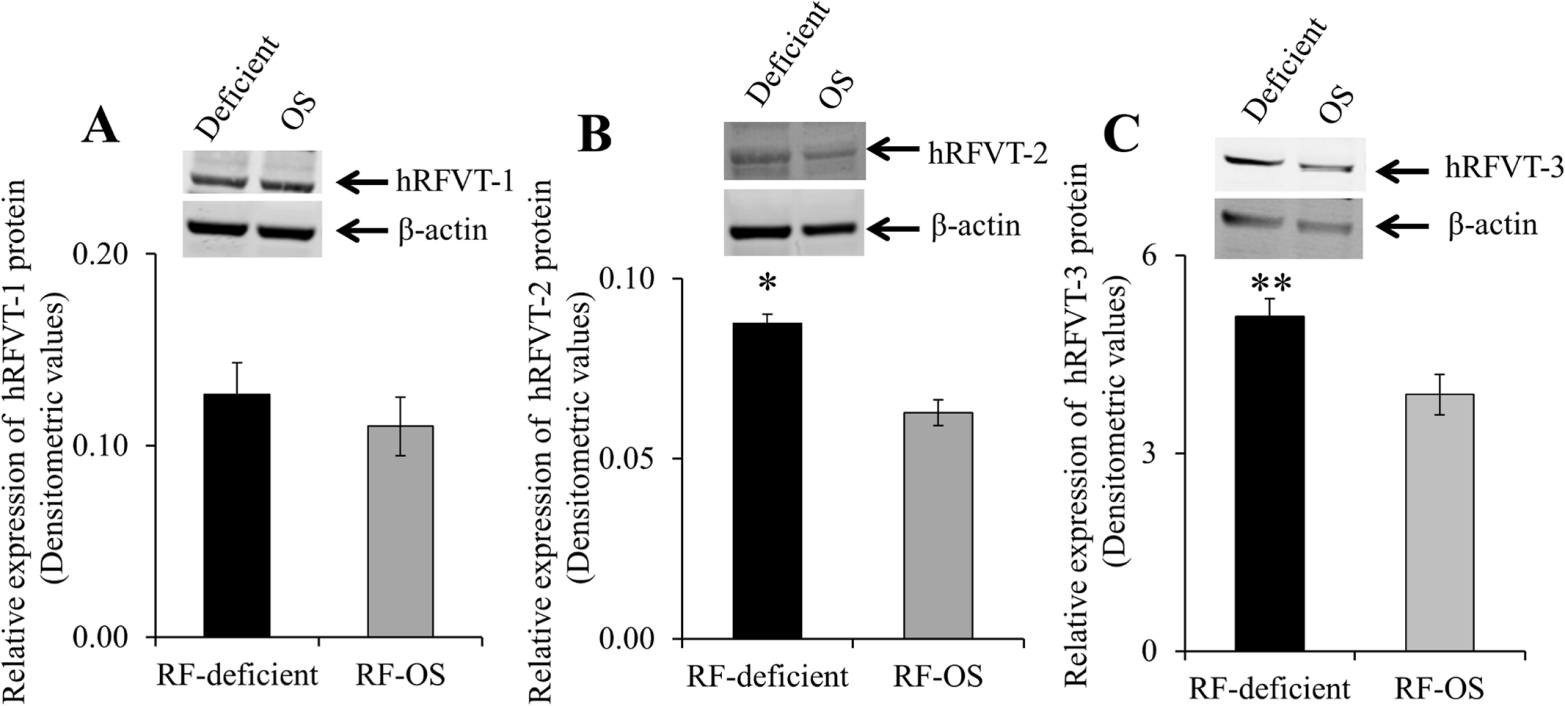
Effect of growing NCM460 cells in RF deficient and OS media on the level of expression of the hRFVT-1, -2 and -3 protein. Cell lysate (60 μg) from NCM460 cells maintained (14 days) in RF deficient and OS conditions were electrophoretically separated as described in “Methods”. Blots were incubated with anti- hRFVT-1 (A), -2 (B) and -3 (C) specific antibodies along with β-actin antibodies (top). Bottom, densitometric values. Data are mean ± SE of 3–4 experiments performed on separately isolated samples.* P < 0.01, ** P < 0.03.

In these studies, we first confirmed that maintaining intestinal NCM460 cells in a RF deficient medium leads to a significantly (P < 0.01) higher RF uptake compared to OS conditions (2,308 ± 399 and 445 ± 39 fmol/mg protein/5 min for RF deficient and OS conditions, respectively). We then determined the effect of RF deficient and OS on the expression of the hRFVT-1, -2 & -3 proteins. Results of the western blot analysis showed a significantly higher level of expression of hRFVT-2 (P < 0.01) and hRFVT-3 (P < 0.03) proteins (with no change in the level of the hRFVT-1 protein) in cells maintained under RF deficient compared to OS condition (Fig 1).

**Fig 2:**
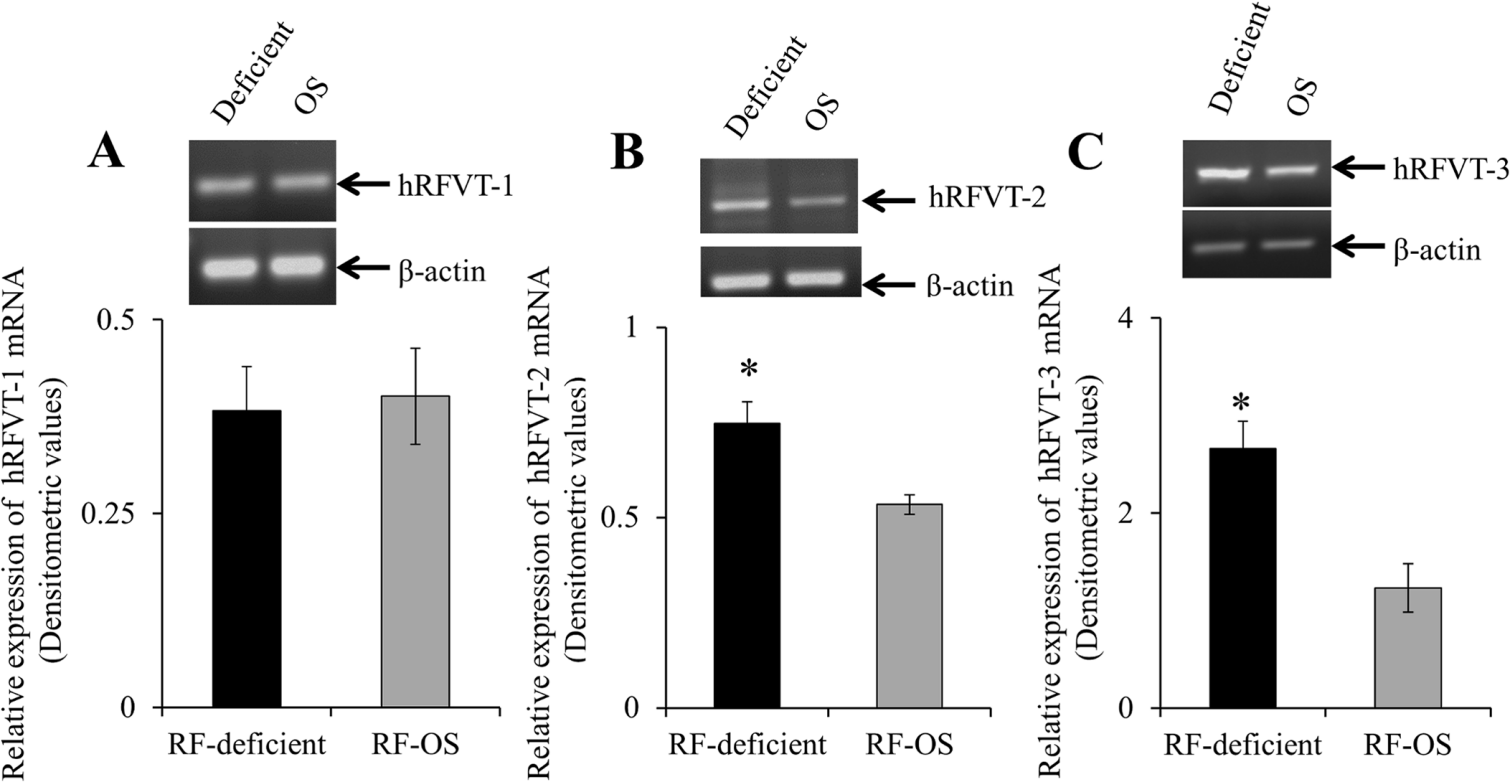
Effect of growing NCM460 cells in RF deficient and OS media on the level of expression of hRFVT-1, -2 and -3 mRNA. RNA was isolated from RF deficient and OS media grown NCM460 cells and cDNA was synthesized and used for semi-quantitative RT-PCR as described in “Methods”. A representative gel image (top) and hRFVT-1 (A), -2 (B), and -3 (C) mRNA expression levels in densitometric values (bottom). Data are mean ± SE of 5–8 experiments performed on separately isolated samples.* P < 0.01.

In other studies, we examined the effect of maintaining NCM460 cells under RF deficient and OS conditions on the expression of hRFVT-1, -2 and -3 mRNAs, with the results showing a significantly (P < 0.01 for both) higher level of expression of hRFVT-2 and -3 mRNA (with no change in the level of the hRFVT-1 mRNA) in cells maintained under RF deficient compared to OS condition (Fig 2).

### Effect of extracellular RF levels on transcriptional activity of the SLC52A3 gene

**Fig 3:**
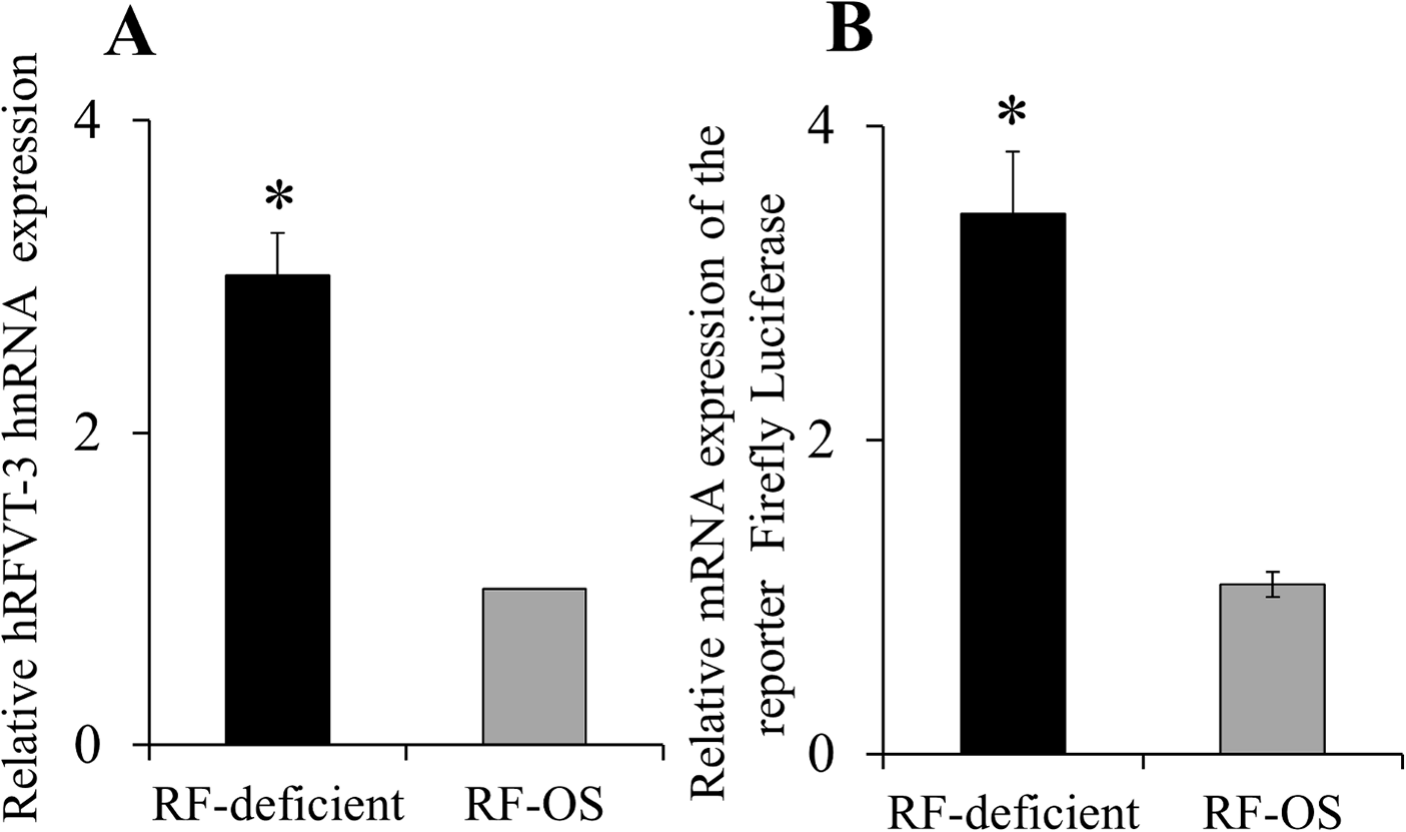
Effect of growing NCM460 cells in RF deficient and OS media on level of expression of the hRFVT-3 (*SLC52A3*) hnRNA and on activity of the *SLC52A3* promoter. (A) RT-qPCR for *SLC52A3* in the RF deficient and OS conditions were performed using gene specific hnRNA primers for *SLC52A3* and β-actin as described in “Methods”. Data were normalized to human β-actin. Data represents the mean ± SE of seven separate sets of experiments. *P < 0.01. (B) Determination of the *SLC52A3* promoter activity was performed as described in “Methods”. Data are mean ± SE of four independent experiments. * P < 0.01.

**Fig 4:**
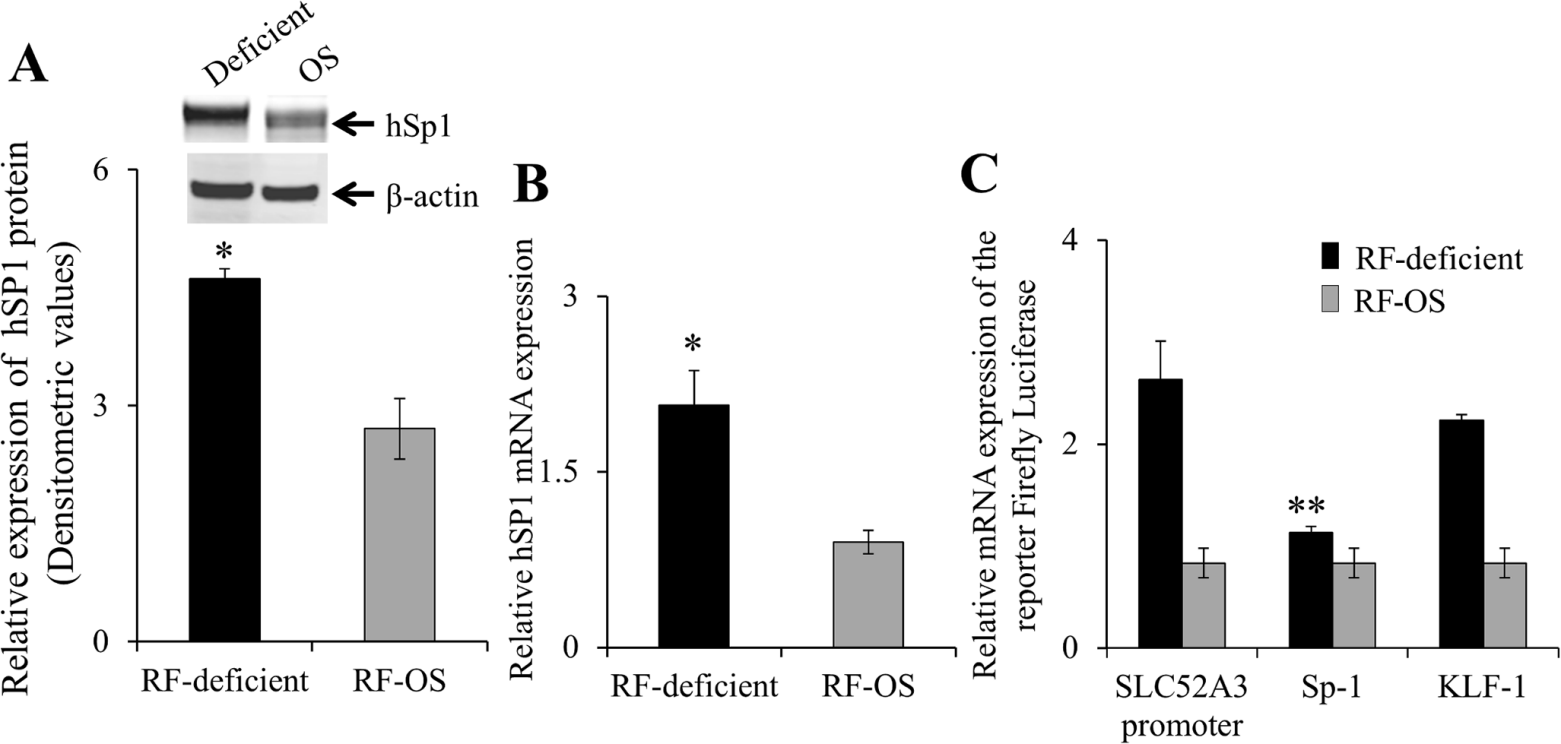
Effect of growing NCM460 cells in RF deficient and OS media on Sp1 protein and mRNA expression levels and effect of mutating Sp1/GC box on induction in activity of the *SLC52A3* promoter in RF deficiency. (A) Western blot analysis was performed using equal amounts (60 μg) of total protein from the cell lysates as described in “Methods”. Blot was probed with the anti-Sp1 polyclonal antibodies. Data represents mean ± SE of four independent sets of experiment are expressed in densitometric values and normalized to the amount of β-actin. Inset is the image of a representative gel. *P < 0.01. (B) The RT-qPCR was performed as described in “Methods” using human Sp1 primers. Data were normalized relative to the human β-actin. Data represents the mean ± SE of five independent sets of experiment *P < 0.01. (C) Activity of the mutated *SLC52A3* promoter assayed as described in “Methods”. Data are mean ± SE of three independent experiments **P < 0.05.

The studies described earlier showing induction of the hRFVT-2 and -3 mRNA suggest that the adaptive regulation of RF uptake by extracellular vitamin level in NCM460 cells may be in part mediated via transcriptional mechanism(s) of the respective gene. To test this possibility, we focused on the *SLC52A3* gene (which encodes the predominant RF transporter (hRFVT-3) in the intestine; [26]), and examined the possible effect of RF deficiency and OS on level of expression of its heterogeneous nuclear RNA (hnRNA; hnRNA is the first product of gene transcription and its level of expression reflects transcriptional activity of the particular gene [36, 37]). The results showed a significantly (P < 0.01) higher level of expression of the *SLC52A*3 hnRNA in cells grown under RF deficient compared to OS conditions (Fig 3A). These findings suggest that the adaptive regulation of RF uptake by extracellular substrate level in NCM460 cells is mediated (at least in part) via an increase in the transcriptional activity of the *SLC52A3* gene. To confirm this suggestion, we examined the effect of RF deficiency and OS on activity of the *SLC52A3* promoter (-199/+8) that we have recently cloned and characterized [39]. The results showed significantly (P < 0.01) higher *SLC52A3* promoter activity in NCM460 cells maintained under RF deficient compared to OS condition (Fig 3B). Since the nuclear factor Sp1 plays an important role in driving the activity of the *SLC52A3* promoter [39], we also examined the effect of RF deficiency and OS on the level of expression of this factor in NCM460 cells. The results showed a significant (P < 0.01) induction in Sp1 expression at both the protein and mRNA level in cells maintained under RF deficient compared to OS conditions (Fig 4A & 4B). To further investigate the role of Sp1 in mediating the induction in *SLC52A3* promoter activity in RF deficiency, we examined the effect of mutating the Sp1/GC cis-element located at position -74/-71 (using TSS as +1) (known to mediate the Sp1 effect on *SLC52A3* promoter activity in intestinal cells [39]). The results showed that mutating this Sp1/GC site leads to a significant (P < 0.05) reduction in the level of induction in *SLC52A3* promoter activity under RF deficient condition (no change in the level of induction in *SLC52A3* promoter activity in RF deficiency was seen when another putative cis-element site, i.e., KLF located at -43/-40 of the *SLC52A3* promoter, that is known to have no role in *SLC52A3* promoter activity was mutated; [39]) (Fig 4C).

**Fig 5:**
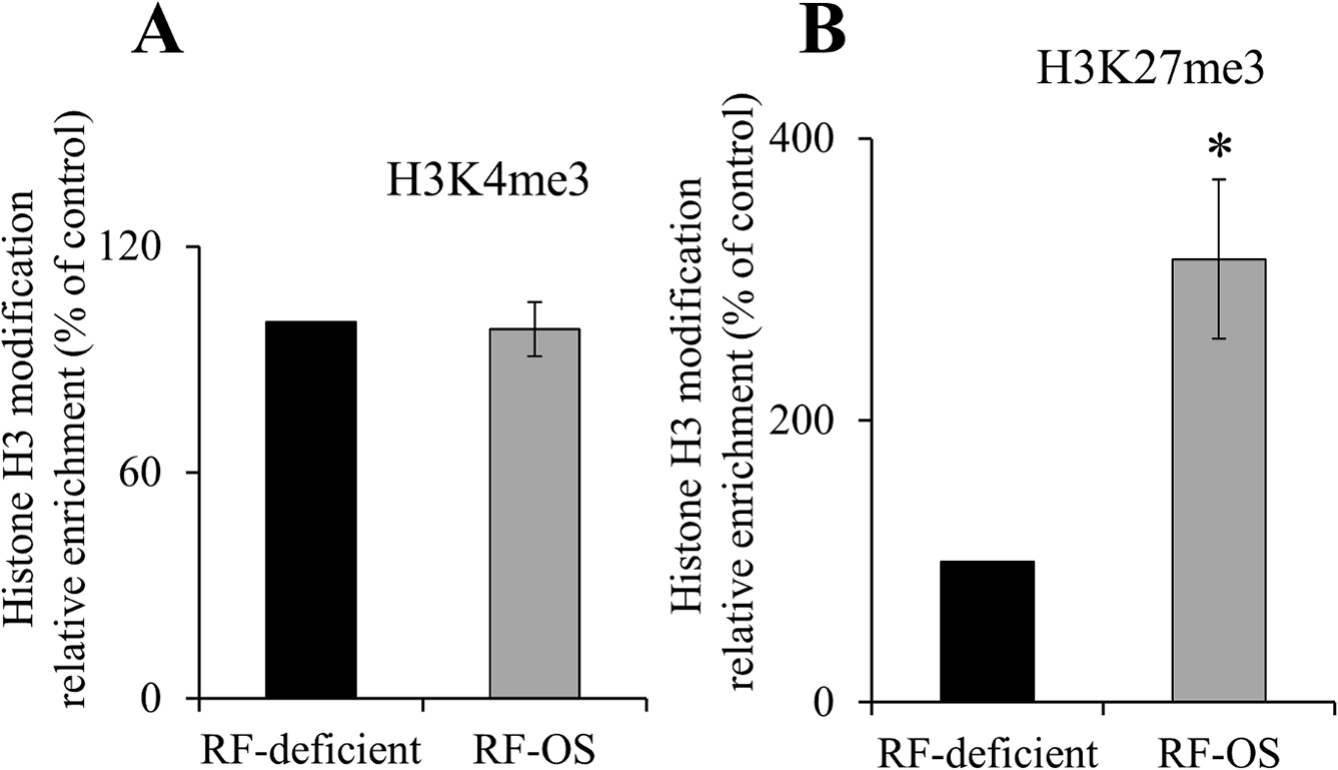
RT-qPCR analysis of H3K4me3 and H3K27me3 in immunoprecipitated DNA fragments on *SLC52A3* promoter of NCM460 cells maintained in RF deficient and OS media. RT-qPCR data showing the ratio of H3K4me3 (A) or H3K27me3 (B) relative to total H3 levels in NCM460 cells maintained under RF deficient and OS media for 14 days. Data are normalized to total input DNA and expressed as mean ± SE of three independent samples. *P < 0.02.

Since promoter activity of a given gene could also be modulated by epigenetic mechanisms (e. g., DNA methylation, histone modification), we also tested whether the induction in *SLC52A3* promoter activity in RF deficiency associated with epigenetic changes. We focused on histone modifications since they play important role in gene activation/silencing [42] [also, subjecting the *SLC52A3* promoter (-199/+8) to Methprimer program [43] did not show putative CpG islands]. Thus, we examined and compared changes in H3 trimethylation of lysine 4 [H3K4me3; a mark of activation (euchromatin)] and in H3 trimethylation of lysine 27 [H3K27me3; a marker of repression (heterochromatin)] in cells maintained in RF deficient and OS conditions. We used ChIP assay utilizing specific antibodies for the H3 modifications followed by RT-qPCR of *SLC52A3* promoter. The results showed that while there was no significant changes in the level of the euchromatin mark (H3K4me3), a significant (P < 0.02) decrease in the activity of the heterochromatin mark (H3K27me3) was observed in cells maintained in RF deficient compared to OS conditions (Fig 5). This raised the possibility that the induction in *SLC52A3* promoter activity observed in RF deficiency may also involve epigenetic mechanism.

### Effect of extracellular substrate level on cell surface expression of the hRFVT-3 in human intestinal epithelial cells

**Fig 6:**
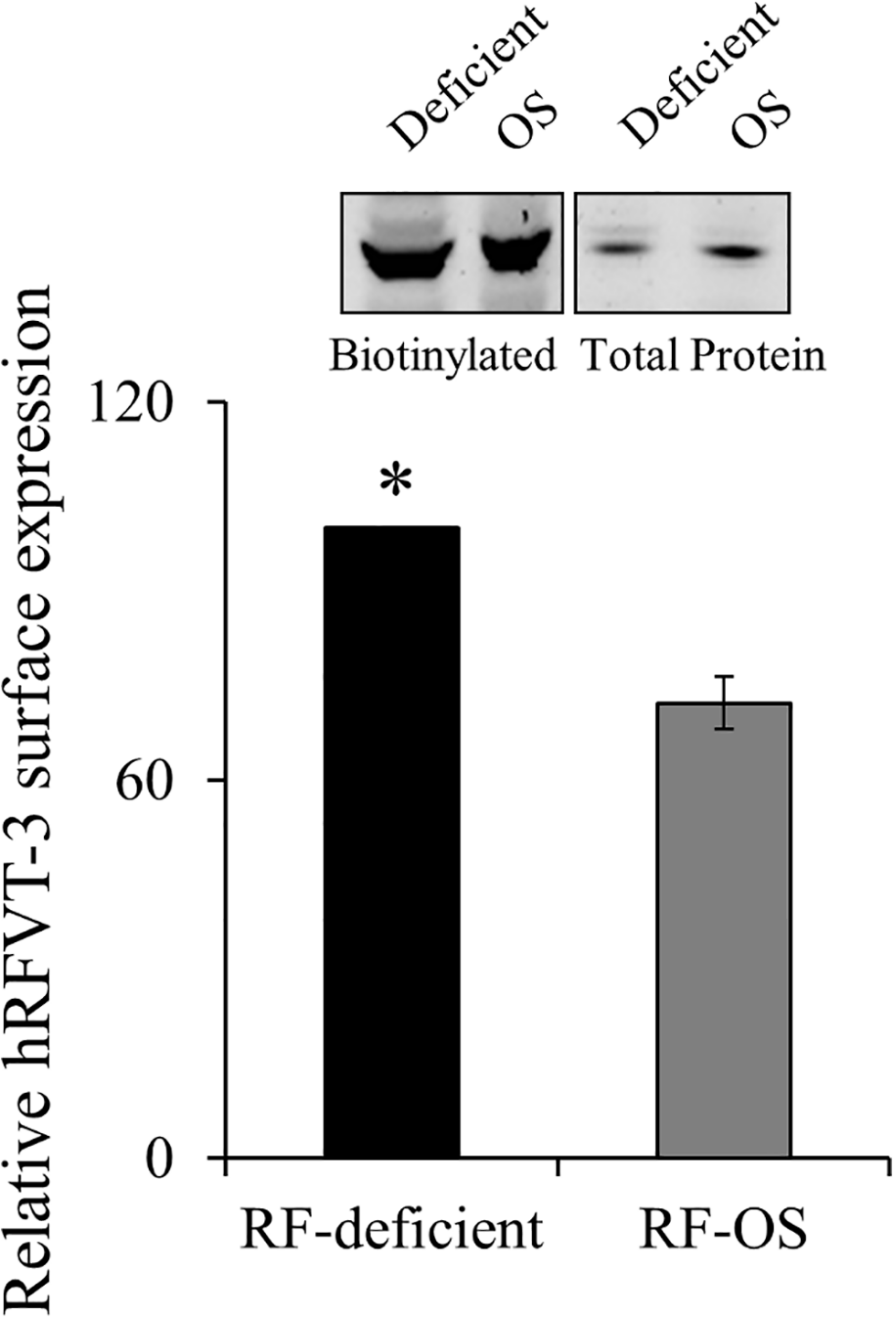
Effect of growing NCM460 cells in RF deficient and OS media on cell surface expression of the RFVT-3 protein. NCM460 cells were maintained (14 days) in RF deficient and OS media followed by performance of biotinylation assay. Equal amount of protein was loaded onto pre-made 4–12% mini-gel, and western blot was performed using anti-hRFVT-3 antibodies. The level of cell surface expression was normalized relative to the total amount of cellular hRFVT-3 protein. Inset shows representative western blot images. Bottom, densitometric values are from mean ± SE of three independent experiments. *P < 0.01.

It has been well established that regulation of a membrane transport event could also involve changes in the level of expression of the transport protein at the cell surface [44–46]. To test whether this also occurs in the case of the adaptive regulation of intestinal RF uptake process by substrate levels, we performed a cell surface biotinylation assay on cells maintained in RF deficient and OS media. The results showed a significantly (P < 0.01) higher level of expression of the hRFVT-3 protein at the cell surface of NCM460 cells maintained under RF deficient compared to those maintained under the OS condition (Fig 6).

## Discussion

Previous studies from our laboratory have shown that the intestinal RF uptake process (and in other cell types) is adaptively regulated by the prevailing extracellular vitamin level [29–33], but nothing is known about the cellular and molecular mechanism(s) involved in this regulation. With the recent availability of molecular and cellular tools, we addressed this issue in this investigation using as a model the human intestinal epithelial NCM460 cells maintained under RF deficient and OS conditions. First, we confirmed the previous findings that RF uptake is significantly higher in cells maintained under RF deficient compared to OS conditions, and extended the study to show that this induction in uptake under the deficient condition is associated with a significant increase in the level of expression of the hRFVT-2 and -3 protein and mRNA (no change in the level of expression of hRFVT-1 was observed). The increase in level of expression of hRFVT-2 and -3 mRNA suggests possible involvement of transcriptional regulatory mechanism(s) affecting the respective genes. Focusing on the predominant and most active RF transport system in the intestine, i.e., hRFVT-3 [26], we tested this possibility by determining the level of expression of the *SLC52A3* hnRNA in cells maintained under RF deficient and OS conditions. The results showed a significantly higher level of hnRNA expression in cells maintained in the former compared to the latter condition confirming the above stated suggestion. Additional support for this suggestion came from the finding of a higher *SLC52A3* promoter activity in cells maintained under RF deficient compared to OS condition. Since previous studies from our laboratory [39] have established an important role for the nuclear factor Sp1 in driving the activity of the *SLC52A3* promoter, we also tested the effect of RF deficient and OS conditions on level of expression of this factor. The results showed a significantly higher level of expression of Sp1 protein and mRNA in cells maintained in RF deficient compared to those maintained in OS medium. To further examine the role of Sp1 in mediating the adaptive response in *SLC52A3* promoter activity by extracellular RF level, we examined the effect of mutating the Sp1 site in the *SLC52A3* promoter on level of induction in promoter activity in RF deficiency. The results showed a marked attenuation in the level of induction in *SLC52A3* promoter activity in RF deficiency, further indicating a role for Sp1 in the adaptive regulatory effect.

As mentioned earlier, epigenetic mechanisms also affect gene transcriptional activity, and thus, we also searched for epigenetic alterations in the *SLC52A3* promoter as a function of extracellular RF level. We focused on histone modifications since it plays important role in regulating transcription (and also because we did not identify putative CpG islands in the cloned the *SLC52A3* promoter). The results showed a significantly higher level of the activity of the heterochromatin H3K27me3 (a marker for repression) in cells maintained in RF OS compared to deficient condition, suggesting possible involvement of epigenetic mechanisms in the adaptive regulation of *SLC52A3* promoter activity by RF levels.

Our investigations have also uncovered a mechanism that may contribute to the induction in RF uptake in RF deficiency via an induction in the level of expression of the hRFVT-3 protein at the cell surface. Regulation of a membrane transport event by such a mechanism is not uncommon and has been observed in case of other substrates and cell types [44–46]. In summary, our investigations show that the adaptive regulation in intestinal RF uptake process is mediated via both transcriptional and post-transcriptional mechanism(s).
